# Childhood Adversities and Later-life Loneliness: Mediating Roles of Health and Intergenerational Relationships

**DOI:** 10.1093/geroni/igaf122.3047

**Published:** 2025-12-31

**Authors:** Ying Xu, Merril Silverstein, Tianqi Zhou, Xiaoyu Fu

**Affiliations:** Syracuse University, Syracuse, New York, United States; Syracuse University, Syracuse, New York, United States; Syracuse University, Syracuse, New York, United States; Syracuse University, Los Angeles, California, United States

## Abstract

Social scientists have long recognized the significance of early-life experiences for later-life well-being. However, the implications of these early-life experiences on loneliness in later life have been largely overlooked. To address this gap, this study investigates the association between childhood adversities (CAs: hunger, lack of medical care, parental loss, and poor health) and later-life loneliness among older adults in rural China, with a focus on the mediating roles of self-rated health and emotional closeness with adult children. Using data from the 2021 wave of the Longitudinal Study of Older Adults in Anhui Province (N = 1,554; males = 810, females = 744), we conducted regression-based mediation analysis for two mediators separately, controlling for respondent age, gender, marital status, educational level, living arrangement, and instrumental activities of daily living. Findings indicate that CAs were associated with greater loneliness (β = 0.473, p < 0.001). Self-rated health partially mediated this relationship (β = 0.4131, p < 0.001), with a significant indirect effect (β = 0.0621). Similarly, emotional closeness with adult children partially mediated this association (β = 0.4291, p < 0.001), with a significant indirect effect (β = 0.0435). These findings underscore the long-term consequences of childhood adversities and highlight the importance of promoting health and strengthening intergenerational relationships as potential interventions to mitigate loneliness among older adults in rural China.

